# 4-[(Hy­droxy)(4-methyl­phen­yl)methyl­idene]isochroman-1,3-dione

**DOI:** 10.1107/S1600536811050975

**Published:** 2011-12-03

**Authors:** Akoun Abou, Abdoulaye Djandé, Adama Saba, Thierry Chiavassa, Rita Kakou-Yao

**Affiliations:** a Laboratoire de Cristallographie et Physique Moléculaire, UFR SSMT, Université de Cocody 22 BP 582 Abidjan 22, Côte d’Ivoire; b Laboratoire de Chimie Bio-organique et Phytochimie, Université de Ouagadougou 03 BP 7021 Ouagadougou 03, Burkina Faso; c Université de Provence, Laboratoire de Spectrométrie et Dynamique Moléculaire, case 542 Avenue Escadrille Normandie Niemen, F-13397 Marseille, Cedex 20, France.

## Abstract

In the title compound, C_17_H_12_O_4_, the six-membered heterocyclic ring adopts a distorted screw-boat conformation. The mol­ecular structure exhibits an *S*(6) ring motif, owing to an intra­molecular O—H⋯O hydrogen bond. In the crystal, weak C—H⋯O contacts generate an infinite chain along the *c* axis. There are also π–π stacking inter­actions between neighbouring isochromanedione benzene rings, with a centroid–centroid distance of 3.755 (1) Å, and C—O⋯π inter­actions with an O⋯centroid distance of 3.964 (2) Å.

## Related literature

For the biological activity of isochromanones, see: Bianchi *et al.*, (2004 ▶); Buntin *et al.* (2008 ▶). For π–π stacking inter­actions, see: Janiak (2000 ▶). For hydrogen-bond motifs, see: Bernstein *et al.* (1995 ▶). For ring puckering parameters, see: Cremer & Pople (1975 ▶).
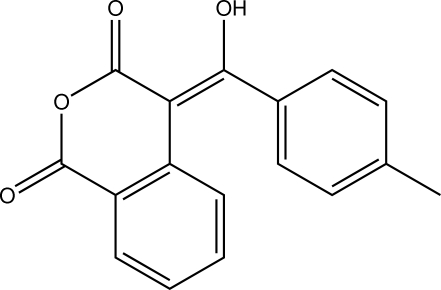


## Experimental

### 

#### Crystal data

C_17_H_12_O_4_
*M* *_r_* = 280.27
Monoclinic, 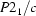

*a* = 15.6767 (6) Å
*b* = 5.9655 (2) Å
*c* = 14.4589 (4) Å
β = 102.961 (1)°
*V* = 1317.74 (8) Å^3^
*Z* = 4
Mo *K*α radiation
μ = 0.10 mm^−1^
*T* = 298 K
0.40 × 0.34 × 0.10 mm

#### Data collection

Nonius KappaCCD diffractometer
12419 measured reflections
3304 independent reflections
2684 reflections with *I* > 2σ(*I*)
*R*_int_ = 0.053

#### Refinement

*R*[*F* ^2^ > 2σ(*F* ^2^)] = 0.057
*wR*(*F* ^2^) = 0.150
*S* = 1.08
3304 reflections
193 parameters
H-atom parameters constrained
Δρ_max_ = 0.20 e Å^−3^
Δρ_min_ = −0.16 e Å^−3^

### 

Data collection: *COLLECT* (Hooft, 1998 ▶); cell refinement: *DENZO*/*SCALEPACK* (Otwinowski & Minor, 1997 ▶); data reduction: *DENZO*/*SCALEPACK*; program(s) used to solve structure: *SIR2004* (Burla *et al.*, 2005 ▶); program(s) used to refine structure: *SHELXL97* (Sheldrick, 2008 ▶); molecular graphics: *PLATON* (Spek, 2009 ▶); software used to prepare material for publication: *SHELXL97*, *publCIF* (Westrip, 2010 ▶) and *WinGX* (Farrugia, 1999 ▶).

## Supplementary Material

Crystal structure: contains datablock(s) I, global. DOI: 10.1107/S1600536811050975/fj2488sup1.cif

Structure factors: contains datablock(s) I. DOI: 10.1107/S1600536811050975/fj2488Isup2.hkl

Supplementary material file. DOI: 10.1107/S1600536811050975/fj2488Isup3.cml

Additional supplementary materials:  crystallographic information; 3D view; checkCIF report

## Figures and Tables

**Table 1:** Hydrogen-bond geometry (Å, °)

*D*—H⋯*A*	*D*—H	H⋯*A*	*D*⋯*A*	*D*—H⋯*A*
O4—H4⋯O3	0.82	1.75	2.485 (2)	148
C7—H7⋯O2^i^	0.93	2.57	3.299 (2)	136

Symmetry code: (i) 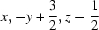.

**Fig. 1.**
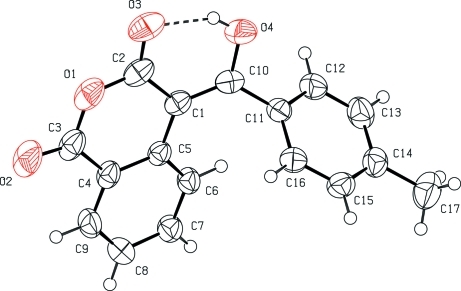
The molecular structure of (I) showing the atomic labeling scheme with displacement ellipsoids drawn at the 50% probability level. H atoms are shown as spheres of arbitary radius. Dashed lines indicate an hydrogen bond.

**Fig. 2.**
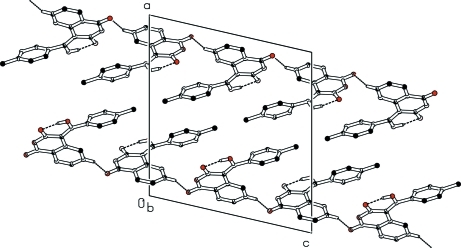
Crystal packing, viewed down the b axis, showing parallel chains along the c direction. Dashed lines indicate hydrogen bonds. H atoms not involved in hydrogen bonds have been omitted for clarity.

**Fig. 3.**
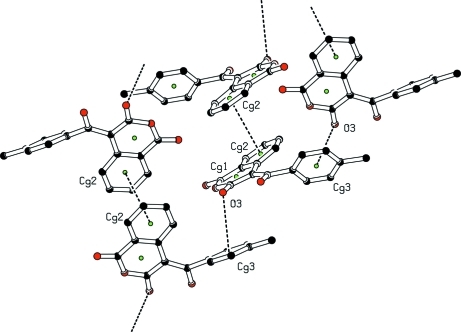
A view of the crystal packing, showing C—O···π and π–π stacking interactions (dashed lines). The green dots are centroids of rings. H atoms have been omitted for clarity.

## supplementary crystallographic information

### Comment

The title molecule is related to the isochromanone derivatives that are generally known as regulators of plant growth (Bianchi *et al.*, 2004). Depending on their chemical structure and concentration they can act either as inhibitors or stimulators in these processes. Some substituted isochromanones isolated from myxobacteria strains were introduced as anti-fungal agents (Buntin *et al.*, 2008).

The structure of the title compound (I) (Fig. 1) consists of two essentially planar benzene rings with the maximum deviations from the best planes of 0.035 (1) Å for atom C6 (benzene ring C4—C9) and 0.008 (2) Å for atoms C12 and C15 (benzene ring C11—C16). An S(6) ring motifs (Bernstein *et al.*, 1995), arising from the intramolecular hydrogen bond O—H···O, generates a planar pseudo six-membered ring (maximum deviation from planarity being ∓0.055 (2) Å for atoms C1 and C10) to result in a tricyclic ring (Fig. 1). The dihedral angles between two benzene rings is 58.99 (8)° and that between the pseudo six-membered ring and benzene rings are 13.75 (8) ° (ring C4—C9) and 53.96 (8)° (ring C11—C16). The heterocyclic ring O1/C1—C5 adopts a distorted screw-boat conformation as judged from the puckering parameters (Cremer & Pople, 1975): Q = 0.0974 (17) Å, θ = 69.6 (1)° and φ = 132.6 (1)°. Furthermore, intermolecular C—H···O hydrogen bonds (Table 1) link molecules into infinite chains along the [001] (Fig. 2).

The supramolecular aggregation is completed by the presence of C—O···π interactions (O3···*Cg3*[*x*,1/2 - *y*,-3/2 + *z*] = 3.964 (2) Å, C2—O3···*Cg3* = 83.89 (12)°, where *Cg3* is the centroid of the benzene ring C11—C16 and π–π stacking between two parallel isochromandione-benzene C4—C9 rings; in the latter, the centroid···centroid distance, (*Cg2*···*Cg2*(-*x*,2 - *y*,-*z*) of 3.755 (1) Å), is less than 3.8 Å, the maximum regarded as relevant for π–π interactions (Janiak, 2000) (Fig.3).

### Experimental

To a solution of *p*-Toluoyl chloride (4.10^-2^ mole) in dried tetrahydrofuran (150 ml), was added dried triethylamine (0.12 mole) and homophtalic anhydride (4.10^-2^ mole) by small portions over 30 min. The mixture was then refluxed for 3 h and poured in 300 ml of chloroform. The solution was acidified with dilute hydrochloric acid until the pH was 2 - 3. The organic layer was extracted, washed with water, dried over MgSO4 and the solvent removed. The crude product was recrystallized from chloroform-hexane (1/1, *v*/*v*) mixture. Yellow crystals of the title compound were obtained in a good yield: 85%; *M*.pt. 387–388 K.

### Refinement

H atoms were placed in calculated positions [O—H = 0.82 Å and C—H = 0.93 (aromatic) or 0.96 Å (methyl group)] and refined using a riding model approximation with *U*_iso_(H) constrained to 1.2 (aromatic) or 1.5 (methyle, O—H) times *U*_eq_ of the respective parent atom.

### Crystal data

**Table:**

C_17_H_12_O_4_	*F*(000) = 584
*M**_r_* = 280.27	*D*_x_ = 1.413 Mg m^−^^3^
Monoclinic, *P*2_1_/*c*	Melting point = 387–388 K
Hall symbol: -P 2ybc	Mo *K*α radiation, λ = 0.71073 Å
*a* = 15.6767 (6) Å	Cell parameters from 12419 reflections
*b* = 5.9655 (2) Å	θ = 2.9–29.0°
*c* = 14.4589 (4) Å	µ = 0.10 mm^−^^1^
β = 102.961 (1)°	*T* = 298 K
*V* = 1317.74 (8) Å^3^	Prism, yellow
*Z* = 4	0.40 × 0.34 × 0.10 mm

### Data collection

**Table:**

Nonius KappaCCD diffractometer	2684 reflections with *I* > 2σ(*I*)
Radiation source: fine-focus sealed tube	*R*_int_ = 0.053
graphite	θ_max_ = 29.0°, θ_min_ = 2.9°
φ and ω scans	*h* = −21→20
12419 measured reflections	*k* = −7→7
3304 independent reflections	*l* = −19→19

### Refinement

**Table:**

Refinement on *F*^2^	Secondary atom site location: difference Fourier map
Least-squares matrix: full	Hydrogen site location: inferred from neighbouring sites
*R*[*F*^2^ > 2σ(*F*^2^)] = 0.057	H-atom parameters constrained
*wR*(*F*^2^) = 0.150	*w* = 1/[σ^2^(*F*_o_^2^) + (0.0625*P*)^2^ + 0.424*P*] where *P* = (*F*_o_^2^ + 2*F*_c_^2^)/3
*S* = 1.08	(Δ/σ)_max_ < 0.001
3304 reflections	Δρ_max_ = 0.20 e Å^−^^3^
193 parameters	Δρ_min_ = −0.16 e Å^−^^3^
0 restraints	Extinction correction: *SHELXL97* (Sheldrick, 2008), Fc^*^=kFc[1+0.001xFc^2^λ^3^/sin(2θ)]^-1/4^
48 constraints	Extinction coefficient: 0.11 (2)
Primary atom site location: structure-invariant direct methods	

### Special details

**Table:**

Geometry. All s.u.'s (except the s.u. in the dihedral angle between two l.s. planes) are estimated using the full covariance matrix. The cell s.u.'s are taken into account individually in the estimation of s.u.'s in distances, angles and torsion angles; correlations between s.u.'s in cell parameters are only used when they are defined by crystal symmetry. An approximate (isotropic) treatment of cell s.u.'s is used for estimating s.u.'s involving l.s. planes.
Refinement. The 2 reflections [(-5 2 1), (2 0 0)] whith (Iobs-Icalc)/Sigma(*I*) superior to 10, are not used in the refinement. The weighted *R*-factor *wR* and goodness of fit *S* are based on *F*^2^, conventional *R*-factors *R* are based on *F*, with *F* set to zero for negative *F*^2^. The threshold expression of *F*^2^ > 2σ(*F*^2^) is used only for calculating *R*-factors(gt) *etc*. and is not relevant to the choice of reflections for refinement. *R*-factors based on *F*^2^ are statistically about twice as large as those based on *F*, and *R*- factors based on ALL data will be even larger.

### Fractional atomic coordinates and isotropic or equivalent isotropic displacement parameters (Å^2^)

**Table:**

	*x*	*y*	*z*	*U*_iso_*/*U*_eq_	
O1	0.85227 (9)	0.2252 (2)	0.70711 (8)	0.0608 (4)	
C5	0.83327 (9)	0.3569 (2)	0.51682 (9)	0.0361 (3)	
C4	0.88171 (10)	0.4900 (3)	0.59015 (10)	0.0420 (4)	
O3	0.77485 (10)	−0.0788 (3)	0.66859 (10)	0.0690 (4)	
O4	0.69333 (9)	−0.1556 (2)	0.50349 (10)	0.0632 (4)	
H4	0.7147	−0.1802	0.5597	0.095*	
C15	0.59439 (11)	0.3609 (3)	0.27099 (12)	0.0484 (4)	
H15	0.5656	0.4979	0.2599	0.058*	
C1	0.78494 (10)	0.1628 (3)	0.54105 (10)	0.0405 (4)	
C6	0.83808 (10)	0.4128 (3)	0.42383 (10)	0.0401 (4)	
H6	0.8110	0.3211	0.3738	0.048*	
C9	0.92667 (11)	0.6800 (3)	0.57079 (12)	0.0514 (4)	
H9	0.9579	0.7670	0.6204	0.062*	
C16	0.63851 (10)	0.3071 (3)	0.36170 (12)	0.0454 (4)	
H16	0.6403	0.4089	0.4108	0.054*	
C11	0.68042 (10)	0.1011 (3)	0.38016 (11)	0.0412 (4)	
C10	0.72372 (10)	0.0383 (3)	0.47841 (12)	0.0441 (4)	
C12	0.67731 (11)	−0.0477 (3)	0.30589 (13)	0.0515 (4)	
H12	0.7043	−0.1870	0.3173	0.062*	
C8	0.92474 (12)	0.7385 (3)	0.47832 (13)	0.0530 (4)	
H8	0.9519	0.8693	0.4648	0.064*	
C3	0.89085 (12)	0.4261 (3)	0.68931 (11)	0.0532 (5)	
O2	0.93083 (11)	0.5246 (3)	0.75773 (9)	0.0782 (5)	
C14	0.59194 (10)	0.2147 (3)	0.19552 (12)	0.0487 (4)	
C13	0.63411 (12)	0.0107 (3)	0.21485 (13)	0.0567 (5)	
H13	0.6334	−0.0896	0.1654	0.068*	
C7	0.88200 (11)	0.6007 (3)	0.40553 (11)	0.0461 (4)	
H7	0.8831	0.6360	0.3432	0.055*	
C2	0.80260 (12)	0.0934 (3)	0.63907 (12)	0.0504 (4)	
C17	0.54283 (14)	0.2771 (4)	0.09711 (14)	0.0719 (6)	
H17A	0.4814	0.2511	0.0913	0.108*	
H17B	0.5634	0.1874	0.0514	0.108*	
H17C	0.5524	0.4327	0.0859	0.108*	

### Atomic displacement parameters (Å^2^)

**Table:**

	*U*^11^	*U*^22^	*U*^33^	*U*^12^	*U*^13^	*U*^23^
O1	0.0781 (9)	0.0731 (9)	0.0303 (6)	0.0141 (7)	0.0104 (5)	0.0079 (5)
C5	0.0390 (7)	0.0374 (8)	0.0311 (7)	0.0060 (5)	0.0063 (5)	−0.0008 (5)
C4	0.0461 (8)	0.0458 (9)	0.0313 (7)	0.0105 (6)	0.0029 (6)	−0.0061 (6)
O3	0.0837 (10)	0.0700 (9)	0.0572 (8)	0.0078 (7)	0.0242 (7)	0.0287 (7)
O4	0.0656 (8)	0.0478 (7)	0.0736 (9)	−0.0084 (6)	0.0102 (7)	0.0191 (6)
C15	0.0448 (8)	0.0428 (9)	0.0566 (10)	0.0044 (7)	0.0092 (7)	0.0052 (7)
C1	0.0452 (8)	0.0419 (8)	0.0357 (7)	0.0064 (6)	0.0116 (6)	0.0047 (6)
C6	0.0435 (8)	0.0450 (8)	0.0306 (7)	−0.0051 (6)	0.0056 (6)	−0.0029 (6)
C9	0.0527 (9)	0.0473 (9)	0.0474 (9)	−0.0001 (7)	−0.0031 (7)	−0.0147 (7)
C16	0.0481 (8)	0.0383 (8)	0.0491 (9)	0.0034 (6)	0.0094 (7)	−0.0026 (6)
C11	0.0382 (7)	0.0355 (8)	0.0482 (8)	−0.0021 (6)	0.0064 (6)	0.0002 (6)
C10	0.0463 (8)	0.0371 (8)	0.0509 (9)	0.0039 (6)	0.0149 (7)	0.0069 (6)
C12	0.0520 (9)	0.0383 (9)	0.0606 (10)	0.0032 (7)	0.0048 (8)	−0.0059 (7)
C8	0.0551 (10)	0.0438 (9)	0.0567 (10)	−0.0094 (7)	0.0053 (8)	−0.0031 (7)
C3	0.0648 (11)	0.0583 (11)	0.0328 (8)	0.0203 (8)	0.0030 (7)	−0.0057 (7)
O2	0.1091 (12)	0.0797 (10)	0.0345 (6)	0.0199 (9)	−0.0081 (7)	−0.0154 (6)
C14	0.0380 (8)	0.0586 (10)	0.0478 (9)	−0.0055 (7)	0.0060 (6)	0.0021 (7)
C13	0.0563 (10)	0.0564 (11)	0.0544 (10)	−0.0008 (8)	0.0059 (8)	−0.0161 (8)
C7	0.0487 (9)	0.0504 (9)	0.0380 (8)	−0.0070 (7)	0.0069 (6)	0.0024 (6)
C2	0.0563 (10)	0.0564 (10)	0.0414 (8)	0.0153 (8)	0.0169 (7)	0.0108 (7)
C17	0.0641 (12)	0.0963 (17)	0.0503 (11)	−0.0034 (11)	0.0024 (9)	0.0090 (10)

### Geometric parameters (Å, °)

**Table:**

O1—C2	1.360 (2)	C9—H9	0.9300
O1—C3	1.392 (2)	C16—C11	1.391 (2)
C5—C4	1.404 (2)	C16—H16	0.9300
C5—C6	1.404 (2)	C11—C12	1.386 (2)
C5—C1	1.469 (2)	C11—C10	1.479 (2)
C4—C9	1.396 (2)	C12—C13	1.383 (3)
C4—C3	1.459 (2)	C12—H12	0.9300
O3—C2	1.229 (2)	C8—C7	1.384 (2)
O4—C10	1.3316 (19)	C8—H8	0.9300
O4—H4	0.8200	C3—O2	1.199 (2)
C15—C16	1.376 (2)	C14—C13	1.383 (3)
C15—C14	1.391 (2)	C14—C17	1.505 (2)
C15—H15	0.9300	C13—H13	0.9300
C1—C10	1.380 (2)	C7—H7	0.9300
C1—C2	1.443 (2)	C17—H17A	0.9600
C6—C7	1.372 (2)	C17—H17B	0.9600
C6—H6	0.9300	C17—H17C	0.9600
C9—C8	1.376 (3)		
			
C2—O1—C3	124.49 (13)	C1—C10—C11	126.59 (14)
C4—C5—C6	116.93 (14)	C13—C12—C11	120.05 (16)
C4—C5—C1	119.11 (13)	C13—C12—H12	120.0
C6—C5—C1	123.87 (13)	C11—C12—H12	120.0
C9—C4—C5	121.33 (14)	C9—C8—C7	119.32 (16)
C9—C4—C3	117.80 (15)	C9—C8—H8	120.3
C5—C4—C3	120.78 (16)	C7—C8—H8	120.3
C10—O4—H4	109.5	O2—C3—O1	115.94 (16)
C16—C15—C14	121.40 (16)	O2—C3—C4	126.9 (2)
C16—C15—H15	119.3	O1—C3—C4	117.10 (15)
C14—C15—H15	119.3	C13—C14—C15	117.73 (15)
C10—C1—C2	116.26 (15)	C13—C14—C17	121.93 (18)
C10—C1—C5	126.02 (13)	C15—C14—C17	120.32 (17)
C2—C1—C5	117.72 (14)	C12—C13—C14	121.58 (16)
C7—C6—C5	121.07 (14)	C12—C13—H13	119.2
C7—C6—H6	119.5	C14—C13—H13	119.2
C5—C6—H6	119.5	C6—C7—C8	121.13 (15)
C8—C9—C4	119.92 (15)	C6—C7—H7	119.4
C8—C9—H9	120.0	C8—C7—H7	119.4
C4—C9—H9	120.0	O3—C2—O1	114.97 (15)
C15—C16—C11	120.24 (16)	O3—C2—C1	125.20 (18)
C15—C16—H16	119.9	O1—C2—C1	119.82 (16)
C11—C16—H16	119.9	C14—C17—H17A	109.5
C12—C11—C16	118.97 (15)	C14—C17—H17B	109.5
C12—C11—C10	120.72 (14)	H17A—C17—H17B	109.5
C16—C11—C10	120.24 (15)	C14—C17—H17C	109.5
O4—C10—C1	121.84 (15)	H17A—C17—H17C	109.5
O4—C10—C11	111.50 (14)	H17B—C17—H17C	109.5
			
C6—C5—C4—C9	5.1 (2)	C16—C11—C12—C13	0.9 (3)
C1—C5—C4—C9	−178.11 (14)	C10—C11—C12—C13	177.89 (16)
C6—C5—C4—C3	−171.19 (14)	C4—C9—C8—C7	−3.5 (3)
C1—C5—C4—C3	5.6 (2)	C2—O1—C3—O2	178.20 (16)
C4—C5—C1—C10	168.82 (15)	C2—O1—C3—C4	−4.3 (2)
C6—C5—C1—C10	−14.7 (2)	C9—C4—C3—O2	3.0 (3)
C4—C5—C1—C2	−11.4 (2)	C5—C4—C3—O2	179.43 (17)
C6—C5—C1—C2	165.14 (14)	C9—C4—C3—O1	−174.25 (14)
C4—C5—C6—C7	−5.5 (2)	C5—C4—C3—O1	2.2 (2)
C1—C5—C6—C7	177.88 (14)	C16—C15—C14—C13	1.2 (2)
C5—C4—C9—C8	−0.7 (2)	C16—C15—C14—C17	179.69 (16)
C3—C4—C9—C8	175.73 (16)	C11—C12—C13—C14	−1.1 (3)
C14—C15—C16—C11	−1.4 (3)	C15—C14—C13—C12	0.1 (3)
C15—C16—C11—C12	0.3 (2)	C17—C14—C13—C12	−178.41 (18)
C15—C16—C11—C10	−176.67 (15)	C5—C6—C7—C8	1.5 (3)
C2—C1—C10—O4	−10.7 (2)	C9—C8—C7—C6	3.2 (3)
C5—C1—C10—O4	169.10 (15)	C3—O1—C2—O3	179.33 (15)
C2—C1—C10—C11	165.94 (15)	C3—O1—C2—C1	−1.8 (2)
C5—C1—C10—C11	−14.3 (3)	C10—C1—C2—O3	8.2 (2)
C12—C11—C10—O4	−52.7 (2)	C5—C1—C2—O3	−171.65 (16)
C16—C11—C10—O4	124.25 (16)	C10—C1—C2—O1	−170.55 (14)
C12—C11—C10—C1	130.41 (18)	C5—C1—C2—O1	9.6 (2)
C16—C11—C10—C1	−52.7 (2)		

### Hydrogen-bond geometry (Å, °)

**Table:**

*D*—H···*A*	*D*—H	H···*A*	*D*···*A*	*D*—H···*A*
O4—H4···O3	0.82	1.75	2.485 (2)	148
C7—H7···O2^i^	0.93	2.57	3.299 (2)	136

Symmetry codes: (i) *x*, −*y*+3/2, *z*−1/2.
